# MALT1 substrate cleavage: what is it good for?

**DOI:** 10.3389/fimmu.2024.1412347

**Published:** 2024-05-28

**Authors:** Bahareh Nemati Moud, Franziska Ober, Thomas J. O’Neill, Daniel Krappmann

**Affiliations:** Research Unit Signaling and Translation, Group Signaling and Immunity, Molecular Targets and Therapeutics Center, Helmholtz Zentrum München – German Research Center for Environmental Health, Neuherberg, Germany

**Keywords:** MALT1, CBM complex, API2-MALT1, protease, substrate cleavage, auto-regulation, cell signaling, RNA metabolism

## Abstract

CARD-BCL10-MALT1 (CBM) signalosomes connect distal signaling of innate and adaptive immune receptors to proximal signaling pathways and immune activation. Four CARD scaffold proteins (CARD9, 10, 11, 14) can form seeds that nucleate the assembly of BCL10-MALT1 filaments in a cell- and stimulus-specific manner. MALT1 (also known as PCASP1) serves a dual function within the assembled CBM complexes. By recruiting TRAF6, MALT1 acts as a molecular scaffold that initiates IκB kinase (IKK)/NF-κB and c-Jun N-terminal kinase (JNK)/AP-1 signaling. In parallel, proximity-induced dimerization of the paracaspase domain activates the MALT1 protease which exerts its function by cleaving a set of specific substrates. While complete MALT1 ablation leads to immune deficiency, selective destruction of either scaffolding or protease function provokes autoimmune inflammation. Thus, balanced MALT1-TRAF6 recruitment and MALT1 substrate cleavage are critical to maintain immune homeostasis and to promote optimal immune activation. Further, MALT1 protease activity drives the survival of aggressive lymphomas and other non-hematologic solid cancers. However, little is known about the relevance of the cleavage of individual substrates for the pathophysiological functions of MALT1. Unbiased serendipity, screening and computational predictions have identified and validated ~20 substrates, indicating that MALT1 targets a quite distinct set of proteins. Known substrates are involved in CBM auto-regulation (MALT1, BCL10 and CARD10), regulation of signaling and adhesion (A20, CYLD, HOIL-1 and Tensin-3), or transcription (RelB) and mRNA stability/translation (Regnase-1, Roquin-1/2 and N4BP1), indicating that MALT1 often targets multiple proteins involved in similar cellular processes. Here, we will summarize what is known about the fate and functions of individual MALT1 substrates and how their cleavage contributes to the biological functions of the MALT1 protease. We will outline what is needed to better connect critical pathophysiological roles of the MALT1 protease with the cleavage of distinct substrates.

## 1 Introduction

**Figure 1:**
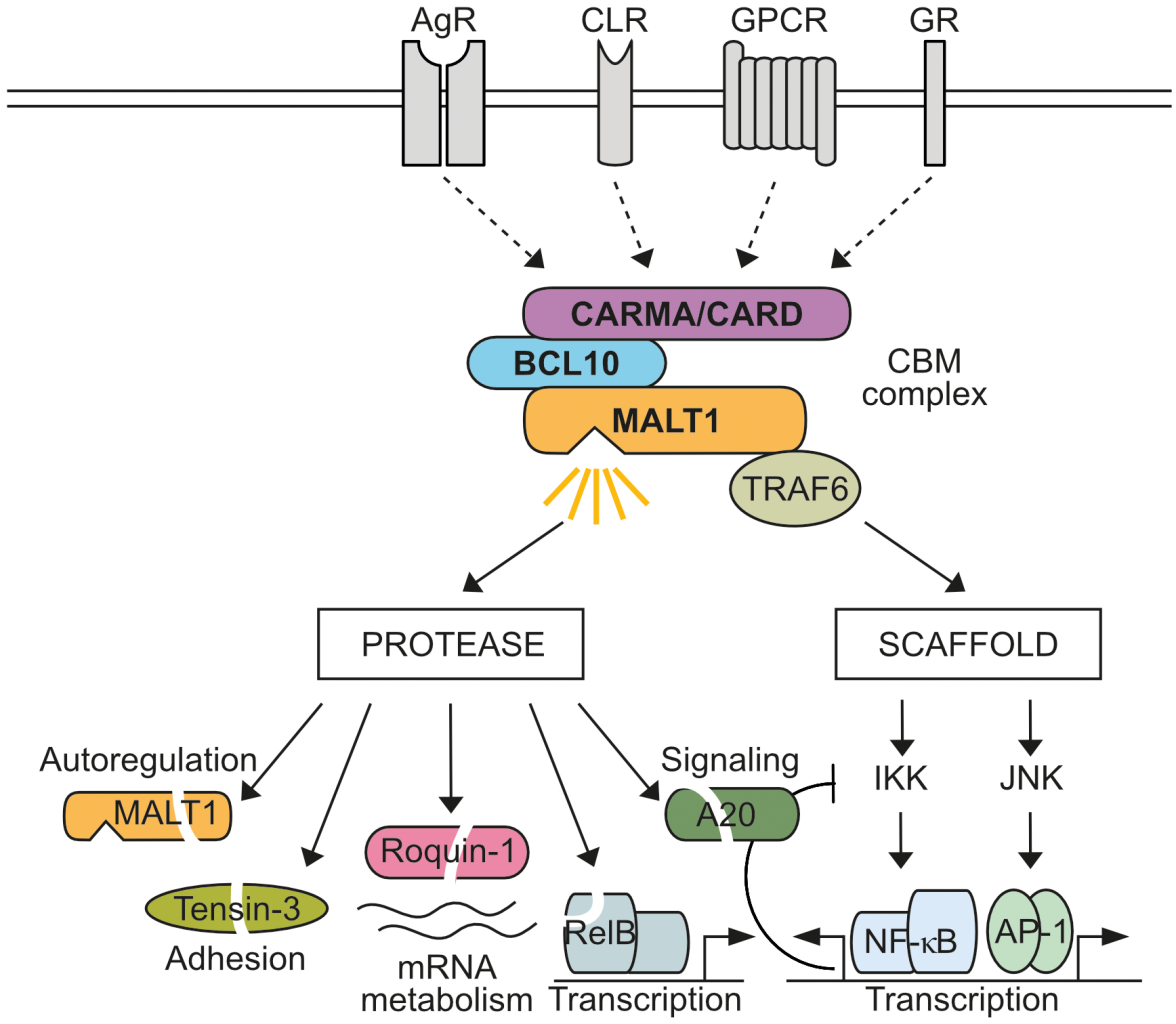
MALT1 bifurcates CBM signaling. Activation of antigen receptors (AR), C-type lectin receptors (CLR), G-protein coupled receptors (GPCR), or growth receptors (GR) induces CARD-BCL10-MALT1 (CBM) complex formation through four distinct CARD scaffolding proteins (CARD9, CARD10, CARD11, CARD14). MALT1 acts as a bifurcation point in the assembled CBM complex. As a non-catalytic scaffold, MALT1 recruits TRAF6 to trigger IκB kinase (IKK)/NF-κB and Jun N-terminal kinase (JNK)/AP-1 signaling, which induces transcriptional reprogramming of cells, including negative auto-regulatory feedbacks. In addition, MALT1 protease is activated, which cleaves several substrates implicated in regulation of signaling, transcription, post-transcriptional mRNA metabolism, CBM auto-regulation and adhesion.

**Figure 2:**
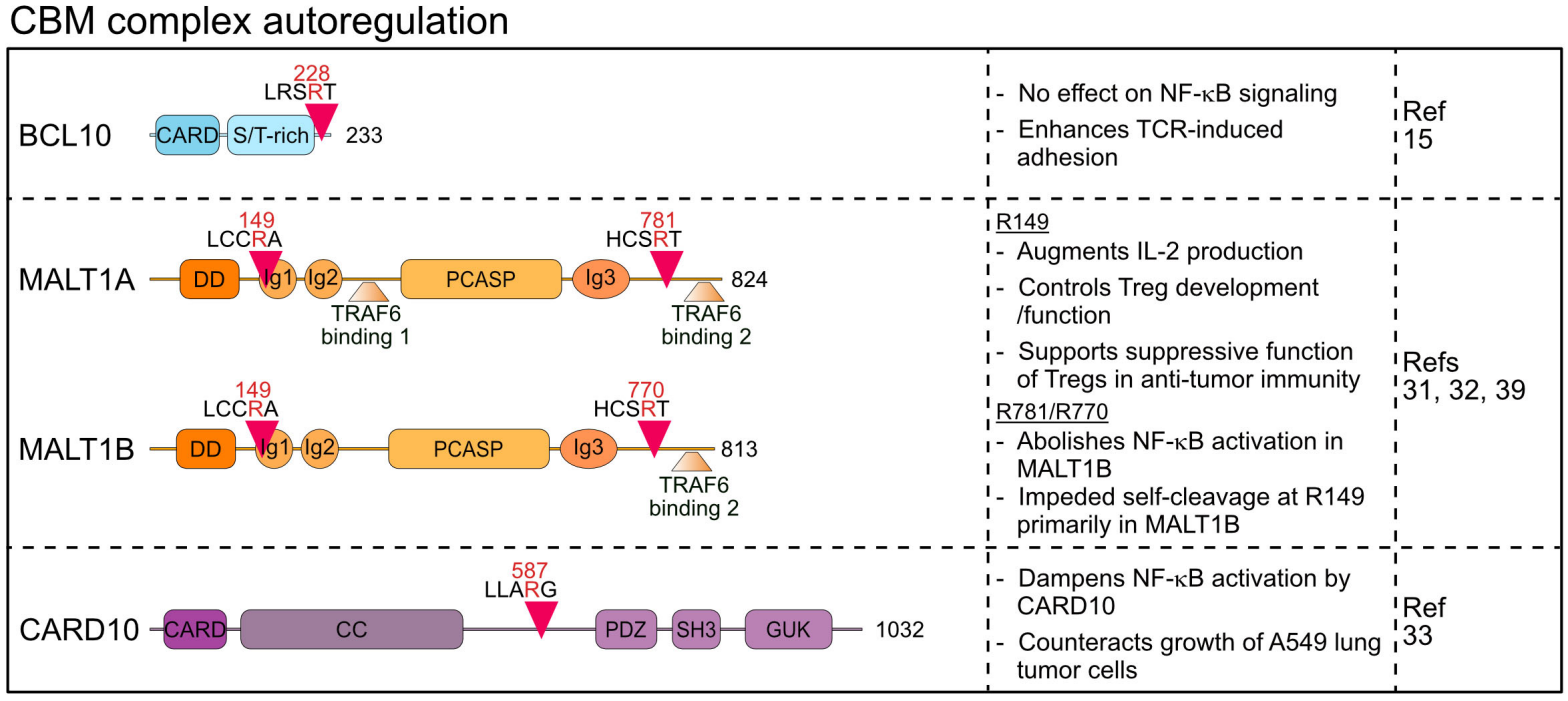
Domain structure, cleavage sites and functions of MALT1 substrates involved in CBM autoregulation. CARD, Caspase recruitment domain; ST-rich, Serine/threonine-rich region; DD, Death domain; Ig, Immunoglobulin; PCASP, Paracaspase; CC, Coiled coil; PDZ, PSD95-Dlg-ZO-1 homology; SH3, Src homology-3; GUK, Guanylate kinase.

Mucosa-associated lymphoid tissue lymphoma translocation protein 1 (MALT1), also known as Paracaspase 1 (PCASP1), is ubiquitously expressed. MALT1 is an integral subunit of various CARD-BCL10-MALT1 (CBM) signaling complexes assembled after ligation of antigen receptors (AR), C-type lectin receptors (CLR), G-protein coupled receptors (GPCR), or growth receptors (GR) (1, 2) (**Figure 1**). With CARD9, CARD10, CARD11 and CARD14, four distinct CARD (caspase recruitment domain)-containing scaffolding proteins can act as seeds to initiate cell- and stimulus-specific assembly of CBM complexes. Most analyses have focused on characterizing the lymphoid-restricted CARD11-containing CBM complex, but similar mechanisms are assumed to govern the activation of other CBM complexes. In lymphocytes, CARD11 acts as a seed to induce B-cell lymphoma/leukemia 10 (BCL10) filament formation via CARD interactions in response to AR engagement (3). In the filaments, the CARD domain of BCL10 also interacts with the N-terminal death domain (DD) of MALT1 in a way that the Immunoglobulin (Ig) and paracaspase domains, as well as the TRAF6 binding motifs (T6BM), protrude from the core filament to form an accessible surface that mediates CBM downstream effector functions (4, 5) (**Figure 2**). Within CBM complexes, both MALT1 scaffolding, and protease functions are activated, and therefore MALT1 acts as a bifurcation point for signaling (**Figure 1**). By recruiting the E3 ligase TRAF6 via two (MALT1 isoform A) or one (MALT1 isoform B) T6BMs, MALT1 scaffolding initiates canonical IκB kinase (IKK)/NF-κB and JNK (c-Jun N-terminal kinase) pathways (6–9). Furthermore, linear ubiquitin chain assembly complex (LUBAC) binds and conjugates linear ubiquitin chains to MALT1 and other CBM subunits, thereby promoting NF-κB signaling by facilitating recruitment of NEMO (NF-κB essential modulator) (10–13). In parallel, the MALT1 protease starts to cleave protein substrates via its paracaspase domain, conferring an enzymatic activity to the CBM complex (14, 15).

Mouse models have been generated to elucidate the physiological functions of MALT1 as well as the specific contributions of its protease and scaffolding activities. Full genetic ablation of MALT1 in mice leads to immune deficiency, resulting primarily from the inability to mount an adaptive immune response after T and B cell antigen receptor (TCR/BCR) ligation (16, 17). Catalytic inactivation of MALT1 in paracaspase dead (PD) mice does not affect scaffolding function and initiation of NF-κB and JNK signaling, but activation of innate and adaptive immune responses is impaired (18–20). However, *Malt1* PD mice develop spontaneous autoimmunity and multiorgan inflammation caused by the developmental loss and functional impairment of suppressive regulatory T (Treg) cells (18–22). In contrast, selective destruction of MALT1 scaffolding in *Malt1* TRAF6-binding mutant (TBM) mice severely compromised CBM downstream signaling but induced severe autoinflammation resulting from unbalanced MALT1 protease activation in conventional effector T cells (8). Thus, the tight balance between catalytic and non-catalytic functions of MALT1 in diverse immune cell subsets is critical to maintain immune homeostasis and allow optimal immune activation. Further, MALT1 protease activation drives survival of lymphoma cells addicted to chronic BCR signaling, activating CARD11 mutations, or expression of the oncogenic API2-MALT1 (BIRC3::MALT1) fusion protein (23, 24). MALT1 protease function is also implicated in the growth of several non-hematologic solid cancers. Potent and selective MALT1 inhibitors have been developed, which are currently undergoing pre-clinical and clinical evaluation for the treatment of MALT1-dependent non-Hodgkin’s lymphoma, but also for depleting suppressive Treg cells in the tumor microenvironment to enhance anti-tumor immunity (24–26). In addition, defective or aberrant MALT1 protease activation has been implicated in human immune errors of immunity caused by germline mutations in CBM components, which have been associated with various immune pathologies such as immunodeficiency, atopy and B cell lymphocytosis (27, 28).

MALT1 belongs to the family of cysteine proteases and the PCASP domain shows a high structural homology to caspases, but in contrast to their aspartate-specific protease activity, MALT1 recognizes a distinct set of substrates cleaved after arginine residues (29). While most initial discoveries of MALT1 substrates relied on serendipity, a bioinformatic workflow confirmed and extended our knowledge to approximately 20-30 MALT1 substrates (30). MALT1 substrates control diverse cellular processes involved in signaling (CYLD, A20, HOIL-1), transcription (RelB), mRNA metabolism (Regnase-1, Roquin-1/2, N4BP1), CBM auto-regulation (CARD10, BCL10, MALT1) and adhesion (Tensin-3), suggesting interdependent and independent functions of the MALT1 protease and scaffold (**Figure 1**). However, despite compelling evidence for an involvement of MALT1 protease activation in maintaining immune homeostasis and driving tumorigenesis, our understanding of the contributions of individual substrate cleavage is still in its infancy. In this review, we will categorize MALT1 substrates according to their main cellular functions and comment on how cleavage of distinct substrates may contribute to the pathophysiological functions of MALT1.

## 2 CBM complex autoregulation by MALT1 cleavage

MALT1 reveals a high degree of feedback regulation, which is mediated by autocleavage as well as cleavage of the CBM core components BCL10 and CARD10 (15, 31–33).

Margot Thome and colleagues were amongst the first to describe MALT1 proteolytic activity by identifying BCL10 as a substrate that is cleaved upon stimulation of antigen and innate immune receptors in lymphocytes and myeloid cells (15). Subsequently, constitutive BCL10 cleavage was also demonstrated in aggressive B cell lymphomas including the activated B cell-type diffuse large B cell lymphoma (ABC DLBCL) and mantle cell lymphoma (MCL), which display constitutive MALT1 protease activity (34–36). In fact, BCL10 is bound in a 1:1 stoichiometry to active MALT1 in the assembled BCL10 filaments (5) and thus, BCL10 cleavage serves as a reliable marker for monitoring MALT1 protease activation in preclinical studies using MALT1 inhibitors (37). MALT1 catalyzes the cleavage of BCL10 at arginine 228, removing only the last five C-terminal amino acids (aa) 229-233 (15) (**Figure 2**). Importantly, this truncation does not affect the ability of BCL10 to bind to MALT1 or to mediate NF-κB activation, and thus BCL10 cleavage is not involved in an autoregulatory feedback circuit that restricts the function of CBM complexes to activate cell signaling. However, MALT1 protease inhibition was shown to impair the antigen-induced adhesion of T cells (15). Since expression of the uncleavable BCL10 R228G mutant weakens fibronectin binding of Jurkat T cells, BCL10 cleavage was suggested to control T cell adhesion in an NF-κB-independent manner mainly through regulating α4β1 integrins. Surface expression of integrin β1 is not controlled by MALT1 or BCL10, indicating that BCL10 cleavage may modulate cytoskeletal changes involved in integrin-ligand binding, but neither the molecular details nor the physiological impact have yet been uncovered. Of note, recent work revealed that MALT1 cleavage of Tensin-3 controls adhesion of B cells, suggesting a broader role of MALT1 in these processes (38) (see chapter 2).

Two studies demonstrated that MALT1 is prone to auto-cleavage at two distinct sites, thereby directly creating auto-regulatory feedback circuits (31, 32). Baens et al. identified auto-proteolysis of MALT1 at arginine 149, creating an N-terminal DD fragment (p19) and a C-terminal fragment (p76) containing Ig1-3 domains, PCASP domain and two T6BMs (31) (**Figure 2**). Since the DD associates with the CARD of BCL10 in the context of BCL10 filaments, the p76 fragment may be released from the core filaments (5). While MALT1 auto-cleavage is clearly detected in studies involving overexpression, reduction of full length MALT1 is not detectable in antigen-stimulated lymphocytes and the cleaved p19 fragment represents only a very small fraction of MALT1 (31). In line, uncleavable MALT1 R149A still mediates TCR-induced NF-κB signaling, but it interferes with optimal induction of NF-κB target genes and production of IL-2 in Jurkat T cells. The underlying mechanism has not yet been defined, but the data suggests that N-terminal auto-cleavage initiates a feedforward pathway involving TRAF6 association to augment transcriptional responses in T cells.

The pathophysiological consequences of MALT1 autocleavage have been investigated in *Malt1* self-cleavage resistant (SR) knock-in mice (39). Like in *Malt1* PD mice, TCR-induced NF-κB signaling in *Malt1* SR mice was normal. Lack of N-terminal MALT1 auto-cleavage did not significantly affect development of conventional effector T cells, but numbers and functions of suppressive regulatory T (Treg) cells was reduced, leading to an improved anti-tumor immune immunity in a syngeneic model. Overall, effects on Treg cells on *Malt1* SR mice were reminiscent but milder when compared to *Malt1* PD mice, suggesting that the MALT1 protease effects on Treg cell functions are partially executed through MALT1 auto-cleavage. More mechanistic work will be required to understand the extent and the impact of MALT1 auto-cleavage in Treg cells and conditional *Malt1* SR mice will be necessary to prove that anti-tumor responses are caused by Treg cell-intrinsic effects.

Ginster and colleagues identified a second auto-processing site at arginine 781 of MALT1, which upon cleavage creates the MALT1 fragment 1-781 that retains all functional domains, but lacks the second C-terminal T6BM2 (aa 804-808) (32). Inducible and chronic C-terminal auto-cleavage of endogenous MALT1 is detected in T cells and lymphoma cells, respectively. Of note, two MALT1 splice isoforms exist and only the longer MALT1A contains the T6BM1 (aa 314-318) and T6BM2, while MALT1B excludes Exon7 encoding for T6BM1 and thus only includes T6BM2 (aa 793-797 in MALT1B) (6) (**Figure 2**). Since TRAF6 binding to MALT1 is essential to mediate NF-κB activation downstream of the CBM complex, cleavage at arginine 781 does not abolish signaling through MALT1A, while it abrogates NF-κB activation by MALT1B (6, 8, 32). Interestingly, self-cleavage in the MALT1 C-terminus and thus reduced TRAF6 binding to MALT1 hampered cleavage at arginine 149, indicating a mutual regulation that governs MALT1 auto-processing (32). Further, TRAF6 association balances MALT1 scaffolding (NF-κB signaling) and protease (substrate cleavage) functions, which is critical for the maintenance of immune homeostasis in mice (8, 40). However, functional analyses of C-terminal auto-cleavage relied on artificial overexpression systems to activate MALT1 protease functions (32). It remains to be determined if inducible C-terminal MALT1 cleavage is functionally relevant *in vivo* and if it potentially only affects certain cell-types such as Treg cells. Of note, a human homozygous missense *MALT1* mutation c.2418G>C (*MALT1A* E806D/*MALT1B* E795D) has been identified, which acts as a hypomorph by selectively destroying the second T6BM and association of TRAF6 to MALT1B but not MALT1A (41). The *MALT1* mutation c.2418G>C causes a complex immune syndrome with combined symptoms of immunodeficiency and autoimmunity, suggesting that the tight control of *MALT1* alternative splicing, and also C-terminal MALT1 auto-cleavage, may tune the balance between immune homeostasis and activation (6, 32, 41).

Besides the shared CBM subunits BCL10 and MALT1, CARD10 (CARMA3) was found to be cleaved by MALT1 (33). CARD10 is widely expressed in non-hematopoietic cells, which contrasts with its homologs CARD11 and CARD14 that display cell-type specific expression in lymphocytes and keratinocytes, respectively. CARD10-containing CBM complexes are activated downstream of distinct GPCRs and receptor tyrosine kinases (RTKs) and control inflammatory responses as well as survival and metastasis of solid cancers (42). CARD10 cleavage by MALT1 occurs in the linker region at arginine 587, thereby disconnecting the N-terminal CARD and CC (coiled-coil) domain from the plasma membrane binding C-terminal MAGUK (membrane-associated guanylate kinase) region (33) (**Figure 2**). Protein kinase C (PKC) activation induces CARD10 cleavage in the lung tumor cell line A549. Since expression of cleavage resistant CARD10 R587A increased IL-6 production and enhanced tumor growth in a mouse xenograft model, CARD10 cleavage by MALT1 may constitute a negative feedback mechanism to limit signaling. However, the N-terminal CARD10 fragment may constitute a signaling competent CBM complex similar to CARD9, in which only a CARD and CC domain is sufficient for its function as an adaptor for innate immune stimulation in myeloid cells (43). Thus, CARD10 is the first MALT1 substrate cleaved explicitly outside the hematopoietic lineage, but further studies must resolve the physiological triggers and downstream functions.

## 3 Impact of MALT1 substrate cleavage on cell signaling and adhesion

MALT1 cleaves several proteins directly connected to NF-κB signaling and transcriptional gene regulation. These include A20/TNFAIP3 (TNFα-induced protein 3), CYLD (cylindromatosis) and HOIL-1 (Haem−oxidized IRP2 ubiquitin ligase 1, also termed RBCK1), which are well-known regulators of NF-κB upstream pathways that control ubiquitination events involved in IKK/NF-κB activation (14, 44, 45). Moreover, Tensin-3 (TNS3) is cleaved by MALT1, which influences B cell adhesion and may thus affect cell responses beyond NF-κB (38).

**Figure 3:**
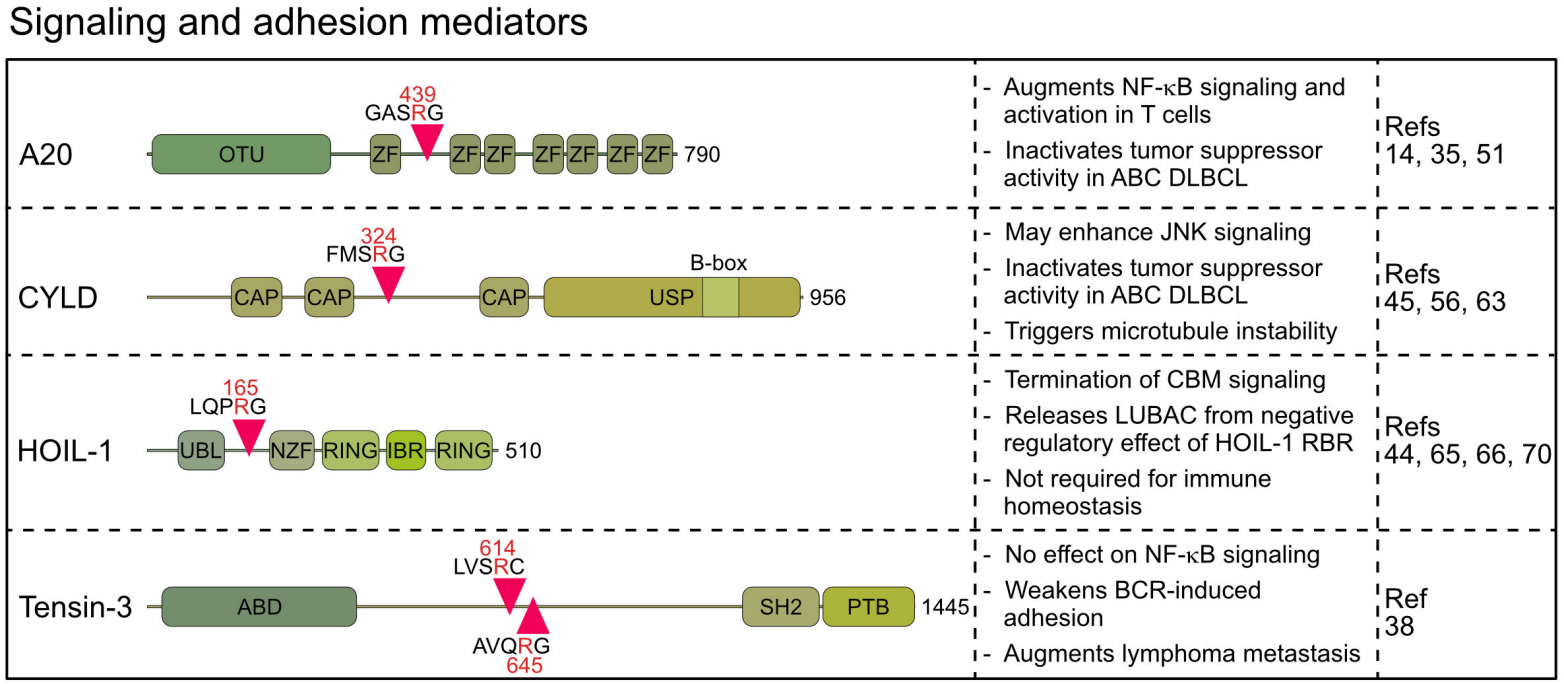
Domain structure, cleavage sites and functions of MALT1 substrates involved in signaling and adhesion. OTU, Ovarian tumor; ZF, Zinc finger; CAP, Cytoskeleton-associated protein; USP, Ubiquitin-specific protease; UBL, Ubiquitin-like; NZF, Npl4 Zinc Finger; RING, Really interesting new gene; IBR, In Between Ring fingers; ABD, Actin binding domain; SH2, Src homology-2; PTB, Phosphotyrosine-binding.

Originally, A20 was discovered as a MALT1 substrate in antigen-stimulated lymphocytes by the Rudi Beyaert lab, which proved that MALT1 is a proteolytic enzyme (14). A20 germline mutations are associated with a wide range of immunological diseases, while somatic mutations inactivate its function as a tumor suppressor in B cell lymphomas (46). Constitutive A20 cleavage by MALT1 is also observed in BCR-addicted and CARD11 mutant aggressive ABC DLBCL, revealing a post-translational mechanism to downregulate A20 (35, 36), which is also observed in MALT1-dependent mantle cell lymphomas (34). Similarly, the oncogenic API2-MALT1 fusion protein, frequently found in mucosa associated lymphoid tissue (MALT) lymphoma, directly catalyzes cleavage of A20 via the MALT1 paracaspase domain (14). A20 has been coined a ubiquitin editing enzyme, because the N-terminal ovarian tumor (OTU) domain preferably hydrolyzes K48- and K63-linked ubiquitin chains, and the C-terminal zinc finger (ZnF) region, specifically ZnF4 and ZnF7, facilitate ubiquitin-conjugation by associating with K63- and M1-linked ubiquitin chains, respectively (47). MALT1 or API2-MALT1 cleave human A20 at arginine 439 between ZnF1 and ZnF2, thereby segregating the N-terminal deubiquitinating activity from C-terminal ubiquitin binding (14) (**Figure 3**). Both A20 fragments are unable to diminish BCL10-induced NF-κB activation, suggesting that A20 is inactivated by MALT1 cleavage. Of note, the cleavage site at arginine 439 is not conserved in murine A20, which is cleaved by MALT1 more C-terminal between ZnF3 and ZnF4. Even though the exact position has not been mapped, the data suggest that MALT1 also inactivates murine A20.

MALT1 can regulate A20 expression in lymphocytes in multiple ways. While MALT1 scaffolding activates NF-κB and transcriptional induction of the *TNFAIP3/A20* gene, MALT1 protease also cleaves and inactivates the RNA binding protein (RBP) Roquin-1 and thereby enhances A20 expression on the post-transcriptional level (48, 49) (see chapter 3). Post-translationally, resynthesized A20 is subjected to MALT1 protease-dependent cleavage and inactivation (14, 50, 51), revealing that MALT1 orchestrates an intricate balance between positive feedforward and negative feedback mechanisms to tightly control A20 amounts. In activated T cells, A20 acts as a negative regulator of CBM-dependent NF-κB signaling by decreasing conjugation of TRAF6 catalyzed K63-linked polyubiquitin chains on MALT1, which is critical for TCR-triggered NF-κB activation (6, 8, 50). While the A20 OTU domain is able to hydrolyze K63-linked ubiquitin chains conjugated to MALT1 *in vitro*, A20 deubiquitinating (DUB) activity is dispensable for counteracting CBM mediated NF-κB activation, which critically relies on ubiquitin binding to A20 ZnF4 and ZnF7 (50, 51). This is in line with results from inflammatory signaling, showing that selective destruction of ubiquitin binding to ZnF4 and ZnF7 in mice phenocopies the autoinflammation and early lethality of complete A20 ablation, while inactivation of DUB activity does not lead to a discernable phenotype (52–54). Likewise, A20 recruitment to the CBM complex and cleavage by MALT1 in T cells relies on intact ZnF4/7 motifs (51). A20 cleavage is impaired in TRAF6/LUBAC double deficient cells, suggesting that A20 binding to M1- and K63-linked ubiquitin chains on BCL10 and MALT1 is necessary for recognition by the MALT1 protease. Interestingly, association of ABIN-1 (A20−binding inhibitor of NF-κB-1, also known as TNIP1) also limits sustained CBM signaling by protecting A20 from MALT1-catalyzed cleavage. Overall, while many mechanistic details have been resolved for how A20 is cleaved by MALT1, the physiological consequences for tuning lymphocyte activation have been poorly defined, and mapping of the exact cleavage site will be necessary to generate A20 cleavage-resistant mice.

With the DUB CYLD, another ubiquitin regulator has been identified as a substrate of MALT1 in activated T cells (45). CYLD is also cleaved by oncogenic API2-MALT1 or chronic MALT1 protease activity in lymphoma cells (34, 45, 55–57). CYLD contains three N-terminal microtubule and ubiquitin binding CAP-Gly domains and a C-terminal ubiquitin-specific protease (USP) domain, which hydrolyzes K63- and M1-linked ubiquitin chains and binds to LUBAC via SPATA2 (58–61). Cleavage of human CYLD takes place at the evolutionary conserved arginine 324 between the second and third CAP-Gly domains, yielding a C-terminal fragment that can still mediate ubiquitin binding via the third CAP-Gly as well as DUB activity and LUBAC binding via the USP domain (**Figure 3**) (59). However, at least in lymphoma cells both CYLD fragments are unstable and degraded by the proteasome in the presence of ibrutinib suggesting that MALT1 cleavage may inactivate CYLD functions (56).

CYLD is critical for counteracting constitutive activation of NF-κB and JNK signaling in T lymphocytes (62) and initial results pointed to a specific role of CYLD cleavage by MALT1 for driving JNK activation (45). However, analyses of primary T and B cells from MALT1 PD mice could not confirm an involvement of MALT1 protease in the initiation of antigen-induced JNK signaling (18–20). Thus, the functional relevance of CYLD cleavage for lymphocyte activation remains enigmatic. Nevertheless, in BCR-addicted ABC DLBCL and MCL low CYLD expression is correlated with poor overall survival and MALT1 cleavage reduces CYLD expression post-translation (56). Thus, in aggressive lymphomas MALT1 releases the negative regulatory impact of CYLD on NF-κB signaling, target gene expression and tumor growth. Evidence for a non-hematopoietic role of CYLD cleavage was derived from endothelial cells stimulated with thrombin, which induces CARD10-dependent MALT1 protease activation through binding to the GPCR protease activated receptor-1 (PAR-1) (63). CYLD binds and stabilizes microtubules via CAP-Gly domains, and accordingly CYLD cleavage triggers microtubule instability, which eventually leads to disruption of the endothelial barrier. Precisely how CYLD fragmentation promotes microtubule disassembly is not clear, but the data suggest that MALT1 protease controls leukocyte migration from the blood vessels to the sites of tissue inflammation in an NF-κB-independent manner. Future analyses must uncover pathophysiological roles of CYLD cleavage in lymphoid and non-lymphoid cells.

While A20 and CYLD counteract multiple NF-κB signaling pathways, the MALT1 substrate HOIL-1 is an integral subunit of LUBAC, which drives NF-κB in response to pro-inflammatory and innate immune stimulation (64). Like other MALT1 substrates, HOIL-1 is cleaved in antigen-stimulated lymphocytes and in malignant ABC DLBCL cells (44, 65, 66). HOIL-1 is cleaved at arginine 165, yielding a short N-terminal fragment that retains the UBL (ubiquitin-like) domain and thus the ability to bind to HOIP (**Figure 3**) (44, 66). The C-terminal fragment of HOIL-1 contains the RBR (RING in between RING) domain, whose catalytic activity can transfer monoubiquitin to LUBAC subunits, which was suggested to impair the ability of LUBAC to conjugate M1-linked ubiquitin chains (67).

LUBAC is required for optimal CBM complex signaling and NF-κB activation after acute TCR/BCR stimulation of lymphocytes as well as chronic BCR survival signaling in ABC DLBCL cells (10, 12, 13, 68). Accordingly, most studies suggest that HOIL-1 cleavage by MALT1 impairs antigen-driven NF−κB activation by decreasing M1-linked ubiquitination, for instance of NEMO, thereby executing a negative feedback loop to terminate signaling (44, 65, 66). However, conflicting results have been found regarding the mechanism, how HOIL-1 cleavage affects antigenic signaling. Overexpression experiments suggested that the HOIL-1 N-terminal fragment retains the ability to mediate NF-κB signaling, whereas the C-terminus counteracts NF-κB activation (66). In contrast, others did not find evidence that the HOIL-1 C-terminal fragment is integrated and regulates LUBAC, but rather that HOIL-1 cleavage leads to reduced LUBAC activity and impaired NF-κB activation (44, 65). In line with reduced LUBAC activity upon HOIL-1 cleavage, skin fibroblasts expressing cleavage-resistant HOIL-1 show slightly augmented NF-κB activation in response to IL-1β (69). However, no phenotype was described in HOIL-1 cleavage-resistant mice, which carry the homozygous R165A mutation generated by CRISPR/Cas9 genome editing (70). In these mice, antigenic signaling, development and numbers of T and B lymphocyte subsets as well as humoral immune responses were normal, arguing that HOIL-1 cleavage does not contribute to the severe pathology observed in MALT1 PD mice. However, the results do not exclude that HOIL-1 cleavage may be relevant in certain pathological settings, for instance in inflammatory responses in the skin caused by CARD14 activating mutations associated with psoriasis (71). Further, the impact of MALT1 cleavage on the roles of the HOIL-1 RBR in impeding LUBAC function by conjugating monoubiquitin on HOIP, SHARPIN and HOIL-1 or in catalyzing ester bonds between ubiquitin and substrates remains unresolved (67, 72).

By integrating protein sequences and functional data, Bell et al. verified known MALT1 substrates and predicted several novel substrates, some of which are involved in cell signaling, including TRAF family member associated NF-κB activator (TANK), TAK1 binding protein 3 (TAB3) and Caspase-10 (CASP10) (30). Cleavage of these three substrates was validated after overexpression of active MALT1 or API2-MALT1 and the cleavage sites were mapped by mutagenesis. TANK cleavage was also detected after PMA/Ionomycin stimulation in B cell lines, but in all cases the functional consequences remain to be defined.

Besides these regulators implicated in NF-κB signaling, Tensin-3 was identified by an unbiased proteomic approach as a MALT1 substrate that is cleaved in ABC DLBCL, MCL and activated B cells (38). Tensins 1–4 comprise a family of proteins that link actin cytoskeleton to integrins, but only Tensin-3 is a MALT1 substrate (38, 73). MALT1 catalyzes cleavage of Tensin-3 at two conserved residues, arginine 614 and 645, separating the N-terminal actin binding domain (ABD) from the C-terminal SH2 and phosphotyrosine binding (PTB) domains, which abrogates the ability of Tensin-3 to bridge actin and integrins (**Figure 3**). Interestingly, Tensin-3 is selectively expressed in primary B cells or B cell lines, but not in T cells, pointing to a specific role in B cell biology and lymphomas (38). To elucidate its function, Tensin-3 non-cleavable (TNS3-nc) mice (R614A/R645A) were generated. TNS3-nc mice did not display gross phenotypic changes and B cell development and responses were normal, but germinal center reactions and antibody responses in immunized mice were mildly reduced. While *Tns3* deficiency did not impact NF-κB or JNK signaling, B cell adhesion to fibronectin-coated plates was impaired. MALT1-uncleavable Tensin-3 showed increased adhesion of human and murine B cells, suggesting a role in B cell homing and migration. In an ABC DLBCL xenotransplant, TNS3 ablation did not affect growth of the primary tumor, but dissemination of tumor cells to the bone marrow and spleen, suggesting that Tensin-3 cleavage may augment lymphoma cell metastasis through modulating B cell adhesion (38). However, the direct impact of Tensin-3 cleavage on metastasis of aggressive lymphoma awaits further investigations and it remains to be seen how other substrates, like BCL10, affect cell adhesion in this context.

## 4 Control of transcriptional and post-transcriptional gene expression by MALT1 substrate cleavage

Besides controlling upstream signaling pathways, MALT1 protease is directly involved in the regulation of transcriptional and post-transcriptional gene expression. The non-canonical NF-κB family member RelB is a substrate of MALT1, which influences NF-κB target gene expression (74). Moreover, with Regnase-1, -2 and -4, Roquin-1 and -2, and N4BP1, six RBPs have been identified as MALT1 substrates, making this class of proteins the largest group of all MALT1 targets so far (30, 48, 75, 76).

**Figure 4:**
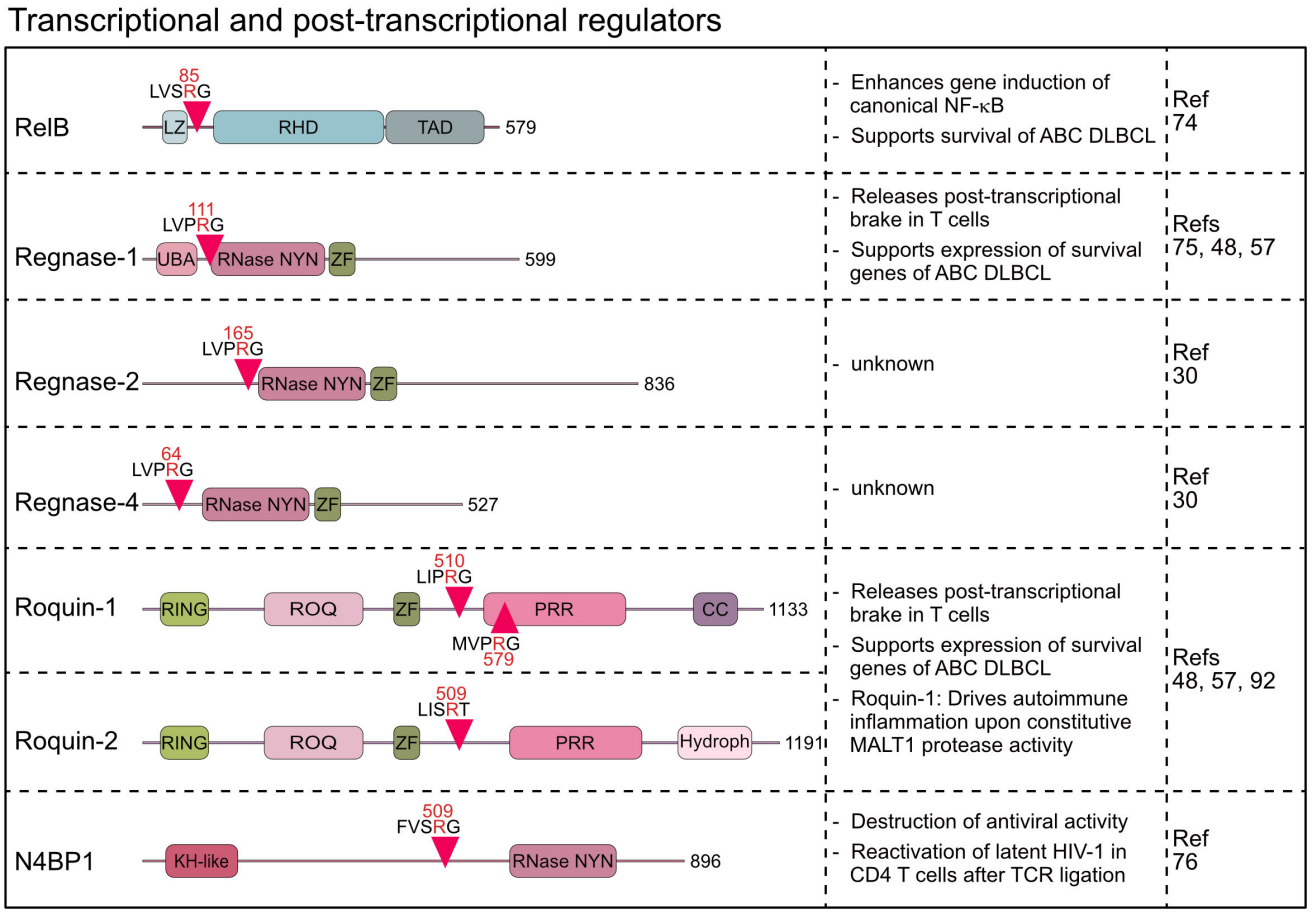
Domain structure, cleavage sites and functions of MALT1 substrates involved in transcriptional and post-transcriptional regulation. LZ, Leucine zipper; RHD, Rel homology domain; TAD, Transactivation domain; UBA, Ubiquitin-associated; RNase-NYN, ribonuclease-N4BP1, YacP-like nuclease; ZF, Zinc finger; RING, Really interesting new gene; PRR, Proline-rich region; CC, Coiled-coil; Hydroph, Hydrophobic region; KH-like, K Homology-like.

RelB was discovered as the third substrate of MALT1, which is cleaved after acute or chronic antigen receptor stimulation in lymphocytes and lymphoma cells, respectively (74). RelB is an NF-κB family member and binds gene regulatory sequences through its N-terminal Rel homology domain (RHD) (77). Like RelA (p65) and c-Rel, RelB contains a transcriptional activation domain (TAD) in the C-terminus and controls target gene expression primarily by dimerizing with nuclear p52, the nuclear product generated by p100/NFKB2 processing in the non-canonical NF-κB pathway. MALT1 cleaves RelB at arginine 85 between the leucine zipper and the RHD, leaving the RHD and TAD intact (74) (**Figure 4**). However, N-terminally truncated RelB is unstable and rapidly degraded by the proteasome, indicating that MALT1 cleavage induces RelB loss-of-function. Functionally, RelB - and even more the uncleavable RelB R85G – was shown to compete with canonical RelA and c-Rel for binding to NF-κB sites on the DNA and to impede gene induction by canonical NF-κB in Jurkat T and ABC DLBCL cell lines. Accordingly, RelB overexpression is toxic in NF-κB-addicted ABC but not GCB DLBCL cells. However, even though earlier studies suggested that RelB interferes with NF-κB activation and RelB deficiency in mice causes autoinflammation, the role of RelB is certainly not limited to its negative impact on canonical NF-κB. For instance, cooperation of RelA and RelB in different thymocyte subsets enhances IL-17 production of γδ T cells and RelB controls homeostatic proliferation of Treg cells (78, 79). In Hodgkin’s lymphoma, RelB/p52 controls gene expression and survival independent of RelA/p50 (80). Thus, it seems unlikely that MALT1 cleavage of non-canonical RelB exerts effects solely by enhancing canonical NF-κB, and further investigations are warranted.

Beyond the impact on signaling and transcriptional responses, the identification of six RBPs as MALT1 substrates indicates a pronounced effect on post-transcriptional gene regulation. The discovery that MALT1 cleaves Regnase-1 (also known as ZC3H12A or MCPIP1) provided first evidence that the MALT1 protease directly affects mRNA metabolism (75). Regnase-1 contains a NYN (Nedd4-BP1, YacP nucleases)-type ribonuclease (RNase) domain, which binds and degrades distinct, primarily translationally active mRNAs, thereby functioning as a brake to prevent uncontrolled translation of transcripts in lymphocytes and macrophages (75, 81–83). Indeed, both T- or B-cell-specific ablation of Regnase-1 causes fatal autoimmune pathologies demonstrating an essential role for Regnase-1 in maintaining immune homeostasis (75, 84). Upon T cell co-stimulation, Regnase-1 is cleaved by MALT1 at arginine 111, just N-terminal to the RNase domain (75) (**Figure 4**). Regnase-1 is degraded after MALT1 cleavage, which with the concomitant decrease of Regnase-1-controlled transcripts such as *ICOS*, *Rel*, *OX40*, *IL2*, and *NFKBIZ* after MALT1 inhibition suggests inactivation of Regnase-1 by MALT1 (48, 75). In mice, conditional overexpression of uncleavable Regnase-1 R111A mutant in CD4 T cells arrested thymic T cell development at the double positive stage causing peripheral lymphopenia (85). However, defective or aberrant MALT1 protease activation does not affect thymic T cell development (8, 19), suggesting that decreases in T cell numbers may be caused by Regnase-1 overexpression and not defective cleavage (85). Recently, the Regnase-1 paralogs Regnase-2 (ZC3H12B) and Regnase-4 (ZC3H12D) were identified as MALT1 substrates upon overexpression of MALT1 or API2-MALT1 fusion protein (30). Similar to Regnase-1, main MALT1 cleavage sites for both RBPs are in the N-terminus at arginine 64 (Regnase-4) and arginine 165 (Regnase-2) (**Figure 4**). MALT1-catalyzed cleavage of Regnase-4 was confirmed in PMA/Ionomycin stimulated B cells, leading to a severe decrease of the full-length protein (30). The functional significance of the parallel inactivation of multiple Regnase family members by MALT1 awaits further investigations. Of note, all four Regnase paralogs are able to downregulate ICOS expression in Regnase-1 knock out T cells, indicating at least partially overlapping functions (86). Thus, MALT1 cleavage of multiple RBPs may elicit more robust effects on post-transcriptional gene regulation.

The wide-ranging effects of MALT1 on mRNA metabolism was further supported by the discovery that the RBPs Roquin-1 (RC3H1) and its paralog Roquin-2 (RC3H2) are also substrates of MALT1 in activated T lymphocytes (48). *Sanroque* mice develop severe autoimmune disease, caused by the missense mutation in the RNA-binding ROQ domain of Roquin-1 (87, 88). Accordingly, Roquin-1 and its paralog Roquin-2 bind transcribed RNAs and control gene expression at the post-transcriptional level by either inducing mRNA decay or translational repression (88–91). MALT1 cleaves Roquin-1 at arginines 510 and 579 and Roquin-2 at arginine 509, generating an N-terminus containing the RNA binding ROQ domain (48) (**Figure 4**). The Roquin-1 N-terminal fragment is either unable (e.g. *Ox40*) or severely impaired (e.g. *ICOS*) in repressing Roquin targets, suggesting that MALT1 cleavage inactivates Roquins (48, 86). *Rc3h1* Mins (MALT1-insensitive) mice, carrying missense mutations destroying both MALT1 cleavage sites in Roquin-1, display normal immune homeostasis under steady state conditions (92). However, it was demonstrated that prevention of Roquin-1 cleavage by MALT1 impairs differentiation of pro-inflammatory Th17 cells and protects from acute experimental autoimmune encephalomyelitis (EAE). Moreover, *Malt1* TBM mice, containing point mutations rendering MALT1 incapable of interacting with TRAF6, succumb to fatal autoimmune inflammation caused by chronic T cell activation resulting from constitutive MALT1 protease activity (8). Blocking Roquin-1 cleavage by crossing *Rc3h1* Mins mice onto the *Malt1* TBM background prevented spontaneous T cell activation and rescued early lethality driven by uncontrolled MALT1 protease function (92). Given the simultaneous processing of multiple RBPs by MALT1, the strong impact of Roquin-1 cleavage alone on these inflammatory phenotypes may be unexpected. However, Roquin-1 and Regnase-1 interact, and share several mRNA targets, including the repression of *Regnase-1* expression as a negative feedback mechanism (48, 82, 86, 91). Thus, due to a high degree of cooperativity between the RBPs, expression of uncleavable Roquin-1 alone seems to affect the entire post-transcriptional program, which may explain the strong amelioration of disease phenotypes. It is tempting to speculate that the homozygous human *MALT1* missense mutation c.2418G>C that abrogates binding of TRAF6 selectively to the MALT1B isoform may also cause the complex immune disorder by augmenting post-transcriptional gene expression through Roquin-1 cleavage (41), but further studies are needed to support that MALT1 protease controls human immunity at the level of mRNA metabolism.

With N4BP1 (NEDD4-binding protein 1) another endoribonuclease has been identified as a MALT1 substrate (76). The RNase domains of N4BP1 and Regnase-1 are related and N4BP1 acts as an IFN-inducible RBP that restricts HIV-1 replication by recognizing and degrading viral RNAs (76, 93). Upon T cell activation, MALT1 cleaves N4BP1 at arginine 509, leading to its inactivation. N4BP1 expression restricts HIV-1 production in latently infected human T cells, and N4BP1 cleavage by MALT1 contributes to the reactivation of HIV-1 after TCR stimulation. These results open a new perspective how MALT1 protease can directly control anti-viral responses. Of note, Regnase-1 has also been established as an HIV-1 restriction factor in resting CD4 T cells (94). Even though it remains speculative, inactivation of various host factors by MALT1 may influence HIV life cycle at different stages, which may also be of therapeutic relevance.

Regnase-1, Roquins and N4BP1 are also subject to MALT1-catalyzed cleavage in lymphomas addicted to aberrant BCR signaling, oncogenic CARD11 or API2-MALT1 (57). By inactivating these RBPs, MALT1 protease augments expression of NF-κB-dependent (e.g. *NFKBIZ*, *BCL2A1* or *IL10*) and NF-κB-independent (e.g. *NFKBID*, *ZC3H12A*) genes. Of note, NFKBIZ/IκBζ expression is controlled by NF-κB and acts as a survival factor in ABC DLBCL (95). On the post-transcriptional level, somatic mutations in the 3’UTR of *NFKBIZ* abrogate binding of Regnase-1, thereby enhancing expression of NFKBIZ/IκBζ in a subset of DLBCL patients (96). Inactivation of Regnase-1 or Roquin-1/2 by MALT1 cleavage serves as an alternative mechanism to release the post-transcriptional brake and induce high expression of oncogenic NFKBIZ/IκBζ in ABC DLBCL (57).

An interesting aspect is that some of the RBPs controlled by MALT1 have also been associated with secondary functions, especially in the regulation of ubiquitination and upstream NF-κB signaling complexes. Roquins contain N-terminal RING domains that function as E3 ligases and catalyze conjugation of various ubiquitin linkages, but it is unclear if and under what circumstances MALT1 cleavage affects E3 ligase activity (97). Upon DNA damage or Toll-like receptor 4 (TLR4) stimulation, Regnase-1 interacts with TANK via the N-terminal ubiquitin association (UBA) domain (98). Regnase-1 recruits the ubiquitin hydrolase USP10 to TANK/NEMO and TANK/TRAF6 complexes, thereby facilitating their de-ubiquitination and termination of NF-κB signaling. Both Regnase-1 and TANK are cleaved by MALT1 (30), but if and how this may affect the activity of upstream signaling complexes has not been resolved. Similarly, N4BP1 associates with NEMO and thereby acts as a potent suppressor of NF-κB activation after innate TLR1/2, TLR7 and TLR9 stimulation in macrophages (99, 100). Interestingly, Caspase 8 (CASP8) cleaves and inactivates N4BP1 in response to TNFα, TLR3 or TLR4 stimulation. CASP8 and MALT1 cleavage takes place at similar positions, suggesting that innate and adaptive immune pathways utilize reminiscent, but independent processes to inactivate N4BP1 (76, 99, 100). If CASP8 and MALT1 affect N4BP1 signaling or RNase functions or both remains to be established.

## 5 API2-MALT1 fusion protein: oncogenic activation by shift in substrate specificity

**Figure 5:**
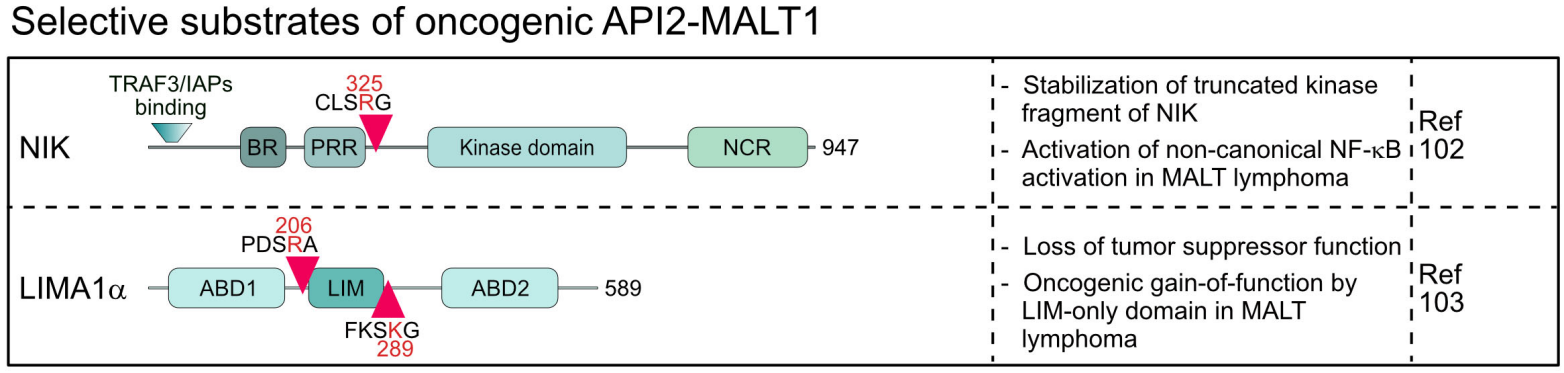
Domain structure, cleavage sites and functions of substrates selectively cleaved by the oncogenic API2-MALT1 fusion protein. BR, basic region; PRR, Proline-rich region; NCR, non-catalytic region; ABD, Actin-binding domain; LIM, LIN-11, Isl-1 and MEC-3.

The oncogenic API2-MALT1 fusion protein is generated by the chromosomal translocation t(11;18)(q21;q21) and activates canonical and noncanonical NF-κB survival signaling in MALT lymphoma (7, 101, 102). MALT1 protease is constitutively activated in the context of the API2-MALT1 and substrates cleaved by MALT1 in the context of an assembled CBM complex are also cleaved by API2-MALT1 (30). However, in the case of NIK (NF-κB inducing kinase) and LIMA1 (LIM domain and actin-binding protein 1) a very peculiar shift in substrate specificity has been observed, because both proteins are substrates of API2-MALT1, but not MALT1 (102, 103). Cleavage of NIK at arginine 325 separates the N-terminal TRAF3 binding region, which destabilizes NIK by recruiting the E3 ligases cIAP1/2, from the protein kinase domain in the C-terminus (102) (**Figure 5**). The truncated kinase fragment of NIK is stabilized, thereby catalyzing chronic IKKα phosphorylation and p100 processing to p52, which drives activation of non-canonical NF-κB. Since NIK is cleaved only by API2-MALT1 and not by endogenous CBM-associated MALT1, non-canonical NF-κB activation is a special feature of MALT lymphomas and not seen in BCR-addicted ABC DLBCL. LIMA1 acts as a tumor suppressor, but cleavage at arginine 206 and lysine 289 surrounding the LIM domain generates a short LIM-only (LMO) fragment that has potent oncogenic functions and enhances B cell lymphomagenesis *in vitro* and *in vivo* (103) (**Figure 5**). So far, LIMA1 is the only substrate that is cleaved after lysine, which is surprising, because neither full length nor paracaspase-only MALT1 accepts a lysine in P1 (104). For NIK and LIMA1, the API2 moiety of the oncogenic fusion is required for substrate recognition, revealing that MALT1 cleavage is context dependent, a circumstance that needs to be considered when searching for novel MALT1 substrates.

## 6 Conclusions and perspectives

The breakthrough discovery of MALT1 protease function in 2008 inspired tremendous research on how this intriguing catalytic activity shapes immune responses. Identification of MALT1 substrates and genetic or pharmacologic MALT1 inactivation have been pursued as the two main strategies to decipher the functions of the MALT1 protease. While the discovery of various substrates untangled cellular pathways and processes controlled by MALT1 cleavage activity, its inactivation demonstrated that MALT1 protease is essential for maintaining peripheral immune tolerance and driving the growth of hematologic and non-hematologic cancers. However, in most cases, it is unclear how cleavage of individual substrates contributes to the pathophysiological functions of MALT1.

It remains an open question, whether we have identified the majority of MALT1 substrates or if we are still looking at the tip of the iceberg. Initial discoveries of substrates primarily relied on serendipity, but recent bioinformatic-guided substrate discovery has identified and confirmed approximately 20 and predicted about 10 more MALT1 substrates (30). However, the algorithm for predicting MALT1 substrates is also based on protein functions, which may create a bias towards known/expected rather than new/unexpected substrates. The relatively loose recognition motif (L-X-S/P-R-G) can be found in many proteins, raising questions about how substrates are recognized and where cleavage occurs in a physiological context. Indeed, the shift in substrate selectivity in the oncogenic API2-MALT1 fusion protein exemplifies the importance of molecular context. It will be crucial to study if different CARDs can facilitate recruitment and targeting of unique MALT1 substrates in a stimulus and cell-type-specific manner, for instance by comparing if CARD14 promotes cleavage of other substrates in keratinocytes than CARD11 in lymphocytes. Further, while there is good evidence that substrates like HOIL-1, BCL10 and A20 are recruited to the CBM complex for cleavage, substrates like RBPs have never been detected at the CBM complex. Using chemical probes, it was shown that active MALT1 is not retained at the CBM complex, but we lack tools to monitor the cellular localization of proteolytically active MALT1, which can also be necessary for the selection of substrates (105). Overall, current studies connecting known substrates to MALT1 protease functions must still be complemented by the discovery of new substrates.

While initial studies mainly focused on analyzing effects of MALT1 substrate cleavage in a cellular context, more recently, *in vivo* functions were explored by generating transgenic mice expressing the non-cleavable substrates MALT1 (R149A), HOIL-1 (R165A), Tensin-3 (R614A/R645A) and Roquin-1 (R510/579A). None of these mice spontaneously develop any severe phenotypes. However, Treg cells were mildly decreased in mice expressing cleavage resistant MALT1, correlating with improved anti-tumor immunity (39). Moreover, cleavage resistant Roquin-1 protects mice from EAE and fatal autoimmune inflammation caused by chronic MALT1 protease activation, revealing that continuous Roquin-1 cleavage, just like its complete ablation, triggers autoimmunity (92). These results emphasize that MALT1 acts as a bifurcation point, with its scaffolding and protease functions coordinating gene induction at the transcriptional and post-transcriptional levels, respectively. Overall, inactivation of these substrates alone is not sufficient to phenocopy the severe effects observed in catalytically inactive *Malt1* PD mice under steady state conditions. However, the effects of individual substrates can modulate responses to immunological challenges. In the future, it will be interesting to see whether combining multiple uncleavable substrates will also affect immune homeostasis.

MALT1 protease is indispensable for Treg cell development and suppressive functions. However, Treg cells provide a paradigm for the difficulties to causally link the effects of the MALT1 protease with the cleavage of specific substrates. Canonical and non-canonical NF-κB activation is required for Treg cell identity and function (106, 107). In addition, CYLD is involved in differentiation of Treg cells (108). Thus, MALT1 cleavage of A20, CYLD or RelB may directly or indirectly enhance NF-κB activation in Treg cells. Alternatively, the atypical IκB protein NFKBID/IκBNS is also needed for Treg development, and IκBNS expression is counteracted at the post-transcriptional level by Roquins, which in turn are cleaved and inactivated by MALT1 (48, 109). However, there are only minor changes in steady state frequencies of Treg cells in Roquin-1 uncleavable mice, arguing that cleavage of Roquin-1 alone is not sufficient to brake peripheral tolerance (92). Indeed, it seems likely that more than one MALT1 substrate is involved in controlling Treg cell development and function. Since it will be highly resource- and time-consuming to intercross mice with various uncleavable substrates, it needs to be explored if *ex vivo* manipulations of Treg-like cell lines or primary Treg cells can help to rationalize which MALT1 substrates are involved in maintaining Treg identity and function (110). Further, identification of relevant MALT1 substrates that drive immune disorders caused by germline mutations in CBM components, so called CBM-opathies, will provide further insights into how the tight control of MALT1 protease activity maintains immune homeostasis (28).

While outstanding work described key roles of MALT1 in immune and oncogenic signaling, we are only beginning to understand what substrates and pathways are responsible for mediating the effects of MALT1 protease. Clinical trials have been initiated to explore beneficial but also potential adverse effects of MALT1 targeting in lymphoma and solid cancers (24). Identifying relevant substrates will be important to better understand the systemic effects of MALT1 inhibition.

## Author contributions

BNM: Conceptualization, Visualization, Writing – original draft, Writing – review & editing. FO: Conceptualization, Visualization, Writing – original draft, Writing – review & editing. TJO: Conceptualization, Writing – original draft, Writing – review & editing. DK: Conceptualization, Funding acquisition, Visualization, Writing – original draft, Writing – review & editing.

## Glossary

**Table:**

ABC DLBCL	Activated B cell-type diffuse large B cell lymphoma
ABD	Actin binding domain
ABIN-1	A20−binding inhibitor of NF-κB-1
API2	Apoptosis inhibitor 2
AR	Antigen receptor
BCL10	B-cell lymphoma/leukemia 10
BCR	B cell antigen receptor
CARD	Caspase recruitment domain
CASP10	Caspase 10
CASP8	Caspase 8
CBM	CARD-BCL10-MALT1
CC	Coiled-coil
CLR	C-type lectin receptor
CYLD	Cylindromatosis
DD	Death domain
DUB	Deubiquitinating enzyme
EAE	Experimental autoimmune encephalomyelitis
GCB DLBCL	Germinal center B cell-type diffuse large B cell lymphoma
GPCR	G protein-coupled receptor
GR	Growth receptor
HOIL-1	Haem−oxidized IRP2 ubiquitin ligase 1
HOIP	HOIL-1 interacting protein
ICOS	Inducible T Cell Costimulator
IFN	Interferons
IKK	IκB kinase
JNK	c-Jun N-terminal kinase
LIMA1	LIM domain and actin-binding 1
LMO	LIM-only
LUBAC	Linear ubiquitin chain assembly complex
MAGUK	Membrane-associated guanylate kinase
MALT1	Mucosa-associated lymphoid tissue lymphoma translocation protein 1
MCL	Mantle cell lymphoma
N4BP1	NEDD4-binding protein 1
NEMO	Nuclear factor-kappa B essential modulator
NFKBID	NF-kappa-B inhibitor delta
NFKBIZ	NFKB inhibitor zeta
NIK	NF-κB inducing kinase
NYN	Nedd4-BP1, YacP-like Nuclease domain
OTU	Ovarian tumor
PAR-1	Protease activated receptor-1
PCASP1	Paracaspase 1
PD	Paracaspase dead
PKC	Protein kinase C
PMA	Phorbol 12-myristate 13-acetate
PTB	Phosphotyrosine binding
RBP	RNA binding protein
RBR	RING in between RING
RHD	Rel homology domain
RTK	Receptor tyrosine kinase
SHARPIN	SHANK associated RH domain interactor protein
SR	Self-cleavage resistant
T6BM	TRAF6 binding motif
TAB3	TAK1 binding protein 3
TAD	Transcriptional activation domain
TANK	TRAF family member associated NF-κB activator
TBM	TRAF6-binding mutant
TCR	T cell antigen receptor
TLR	Toll-like receptor
TNF	Tumor necrosis factor
TNFAIP3	TNFα-induced protein 3
TNS3	Tensin-3
TNS3-nc	Tensin-3 non-cleavable
TRAF6	Tumor necrosis factor receptor associated factor 6
Treg	Regulatory T cells
UBA	Ubiquitin associated
USP	Ubiquitin-specific protease
ZnF	Zinc finger
